# Editorial: The role of foods, diet, and dietary patterns in the prevention and management of diabesity

**DOI:** 10.3389/fnut.2025.1632666

**Published:** 2025-06-23

**Authors:** Luciana Neri Nobre, Elizabethe Adriana Esteves

**Affiliations:** ^1^Program in Sciences of Nutrition, Nutrition Department, Universidade Federal dos Vales do Jequitinhonha e Mucuri, Diamantina, Brazil; ^2^Program in Health Science, Universidade Federal dos Vales do Jequitinhonha e Mucuri, Diamantina, Brazil

**Keywords:** diabesity, foods, diet, dietary pattens, prevention

Diabesity is a term used to describe the combination of obesity and type 2 diabetes, which are closely related. The term “diabesity” was coined in the 1970s by Sims et al. (1) to describe the pathophysiological link between type 2 diabetes and excess body weight. Obesity or high visceral adiposity leads to insulin resistance and type 2 diabetes. In contrast, type 2 diabetes can affect metabolism, contributing to weight gain, an essential mechanism for developing this problem.

Diabesity is a public health issue of growing concern, with significant implications for the cardiovascular health of its sufferers, in addition to other health problems. Thus, optimal treatment strategies for this disease should also include optimal diabesity management (2–4). In this context, this Research Topic highlights the significant role of dietary interventions in preventing and managing diabesity, presenting evidence from eight diverse studies. In this electronic collection, eight articles address some of the abovementioned aspects.

In the first study (Bahari et al.), a meta-analysis evaluated the effects of okra (*Abelmoschus esculentus*) consumption on cardiometabolic risk factors in subjects with prediabetes and diabetes. The authors analyzed nine randomized clinical trials (RCTs) from 1,339 screened articles. They also used random-effects models to calculate weighted mean differences (WMD) for outcomes. They found that okra consumption improves lipid profiles (TC, LDL) and glycemic control (FBG, HbA1c), especially at doses ≤ 3,000 mg/day, but recommended further research to determine the optimal dose and duration of okra intervention for maximal benefits (Tang et al.).

In the second study (Zhu et al.), the authors investigated whether dietary decanoic acid (DDA), a medium-chain fatty acid, influences diabetes risk. Data was acquired from the National Health and Nutrition Examination Survey (NHANES, 2005–2016), with 11,477 adults categorized by diabetes/prediabetes status. They found that higher dietary DDA intake may protect against diabetes progression in prediabetic individuals, particularly those with higher education. However, DDA did not significantly affect prediabetes risk.

The third study (Tang et al.) assessed the global burden of T2DM linked to diet in elderly populations. The data source was from the Global Burden of Disease Study (GBD) 2021, which included 204 countries, adults ≥65 years. They concluded that diet contributes significantly to T2DM burden in elderly adults, with unequal impacts across regions/sexes. They also recommended targeted interventions to reduce processed meat/sugar, promote whole grains/fruits, improve healthcare access, especially in low/middle SDI regions, and personalized nutrition for aging populations.

The fourth study (Tian et al.) evaluated the impact of low-carbohydrate diets on the metabolic profiles of patients with diabetes, aiming to guide clinical practice. The authors identified that low-carbohydrate diets significantly improved HbA1c, fasting blood glucose, and triglyceride levels and increased HDL-c without affecting LDL-c and total cholesterol. In addition, this type of diet favored weight loss, reduction in body mass index (BMI), diastolic blood pressure, and abdominal circumference. Thus, low-carbohydrate diets can improve glycemic control and lipid profile in patients with diabetes, justifying their consideration in managing type 2 diabetes (T2DM). However, due to the variability of carbohydrate restrictions, the authors reinforce the need for further research to standardize dietary guidelines and evaluate long-term effects.

The fifth study (Yang H. et al.), a cross-sectional analysis of four waves of data from the National Health and Nutritional Examination Surveys (NHANES) between 2013–2020, assessed the association between vitamin B2 intake and diabetes (DM) among adults in the US. It was found that vitamin B2 intake was significantly associated with DM in women but not in men. Each standard deviation increase in vitamin B2 intake was associated with a 19% reduction in the odds of DM in women. It is noteworthy, however, that this study did not identify the type of diabetes, and type I diabetes is unrelated to dietary intake or behavior.

The sixth study (Yang S. et al.) aimed to predict the effectiveness of inulin treatment for T2DM using a machine learning model (XGBoost-SHAP model). In this study, from 758 T2DM patients, 477 (62.93%) achieved HbA1c < 6.5% post-inulin treatment. LASSO regression identified six key predictors of treatment success. They used an XGBoost algorithm, which was evaluated by performance metrics (accuracy, specificity, and positive/negative predictive values) and interpretability (SHAP analysis ranked feature importance). The XGBoost-SHAP model successfully identified T2DM patients likely to respond to inulin, enabling personalized nutrition therapy. The learning model could guide clinicians in selecting candidates for the inulin intervention. However, they warned that the model must be validated in diverse populations, and exploring additional biomarkers (e.g., gut microbiota profiles) is necessary.

The seventh study (Wei and Yu), developed with data from NHANES participants from 2005 to 2018, investigated whether there is an association between cardiometabolic indexes (CMI) and frailty index (FI) in patients with DM. CMI is a new marker of metabolic status and is calculated by the formula: CMI = [WC (cm)/height (cm)] × (TG/HDL-c), and the FI was evaluated. The FI considered seven domains and 49 items, which included cognition, dependence, depressive symptoms, comorbidities, physical performance and anthropometry, hospital use and access to care, and laboratory tests. The authors found that the higher the CMI, the higher the FI in participants with DM. This study also did not classify the type of diabetes; however, CMI is an indicator that can reflect both obesity and lipids and can be used to identify DM (5) and elevated glycated hemoglobin (6). Thus, this study indirectly indicated that CMI reflects diabesity in participants, and having this problem favors a higher rate of frailty. The authors conclude that maintaining a healthy, low-fat dietary pattern and adequately controlling blood lipid levels can reduce the risk of frailty in people with DM.

The eighth study (Landa-Anell et al.) discusses the prevalence and impact of nutritional diagnoses in individuals with type 2 diabetes (T2D). The study used the Nutrition Care Process (NCP) model to assess and diagnose patients, finding that the most common nutritional diagnosis was excessive energy and carbohydrate intake. The study analyzed data from 2,050 patients and found that dietary diagnoses in the intake domain, followed by the behavioral/environmental and clinical domains, are highly prevalent in people with T2D. These diagnoses are associated with poorer metabolic control, higher BMI, and increased energy and carbohydrate intake. The research emphasizes the importance of timely identification of nutritional problems to improve disease control and promote a healthy lifestyle.

While these findings advance our understanding of diabesity management, further research is needed to standardize dietary recommendations, explore long-term effects, and validate interventions across diverse populations. Addressing diabesity requires a multifaceted approach, integrating evidence-based nutrition, personalized medicine, and public health strategies to mitigate its growing burden. By prioritizing dietary modifications and early intervention, healthcare providers can better support individuals in achieving metabolic health and reducing diabesity-related complications.
